# Both morphological and molecular data are crucial for describing new species — a case study on the genus *Oreocharis* (Gesneriaceae)

**DOI:** 10.3389/fpls.2025.1641488

**Published:** 2025-09-08

**Authors:** Zhi Xie, Nana Peng, Genglin Xie, Fang Wen, Hanghui Kong

**Affiliations:** ^1^ Key Laboratory of National Forestry and Grassland Administration on Plant Conservation and Utilization in Southern China, South China Botanical Garden, Chinese Academy of Sciences, Guangzhou, China; ^2^ South China National Botanical Garden, Guangzhou, China; ^3^ University of Chinese Academy of Sciences, Beijing, China; ^4^ College of Forestry and Landscape Architecture, South China Agricultural University, Guangzhou, China; ^5^ Guangxi Key Laboratory of Plant Conservation and Restoration Ecology in Karst Terrain, Guangxi Institute of Botany, CAS, Guilin, China; ^6^ National Gesneriaceae Germplasm Resources Bank of GXIB, Gesneriad Committee of China Wild Plant Conservation Association, Gesneriad Conservation Centre of China (GCCC), Guilin Botanical Garden, CAS, Guilin, China

**Keywords:** morphology, molecular analyses, *Oreocharis*, transcriptome data, phylogeny

## Abstract

The concept and definition of species are fundamental in taxonomy and evolutionary biology. Traditionally, new species have been described primarily based on the morphological species concept (MSC). However, environmental factors may drive morphological convergence among distantly related species. In addition, widespread hybridization, gene flow, and incomplete lineage sorting (ILS) among closely related taxa can further obscure phylogenetic relationships and hinder accurate species delimitation. In the current study of the genus *Oreocharis* Benth. (Gesneriaceae: Didymocarpoideae), we built upon a broad phylogenomic framework established in previous work. We incorporated transcriptome data of six specimens from Sichuan, China, and conducted detailed morphological comparisons and phylogenomic analyses. The integrative results reveal that these specimens represent a previously unrecognized lineage, which we formally describe here as *Oreocharis yanbianensis* Z. Xie & H. H. Kong, sp. nov. Our study demonstrates the importance of combining morphological and genomic data to improve the accuracy and robustness of species discovery and classification, especially in morphologically variable plant groups.

## Introduction

Understanding the concept of species is crucial for the study of biodiversity, species classification, and unraveling the complexities of life on Earth (Michener et al., 2001). Over the years, scientists and taxonomists have proposed various species concepts to define and distinguish different groups of organisms (Zhou and Yang, 2011; Hong, 2016). Among the most widely recognized concepts are the Biological Species Concept (BSC, Darwin, 1859; Sokal and Crovello, 1970; Futuyma, 2013; Futuyma and Kirkpatrick, 2017), morphological species concept (MSC, Simpson, 1943), Ecological Species Concept (ESC, Valen, 1976), Phylogenetic Species Concept (PSC, Cracraft, 1983), Cohesion Species Concept (CSC, Templeton, 1989), Recognition Species Concept (RSC, Paterson, 1985; 1992), Evolutionary Species Concept (EvSC, Simpson, 1951; 1961; Wiley, 1992), and General Lineage Species Concept (GLSC, de Queiroz, 1999). Each of these concepts offers unique criteria for defining species, reflecting the diversity of life forms on our planet (Hong, 2016).

The morphological species concept (MSC), which is based on observable morphological characters, has been a favored species concept among botanists for describing new plant species (Mishler, 1985). The morphological characteristics of plants serve as valuable indicators for species identification (Wesselink and Kuiper, 2008). However, there are limitations to relying solely on morphological features in species delimitation (Wiens, 2007). Environmental influences, such as habitat conditions and selective pressures, can cause convergent evolution, leading to similar morphological traits in species that are not closely related (Losos, 2011; Stern, 2013; Hodge et al., 2025). This convergence can result in misinterpretations of species relationships, as species with similar morphological characters might be mistakenly grouped together (Bogarín et al., 2019; Karbstein et al., 2024). A prominent example of such challenges is found within the Gesneriaceae, which includes the genus *Oreocharis*, due to its high morphological variability and frequent misidentification.

*Oreocharis* Benth. is a large genus in the tribe Trichosporeae (Gesneriaceae: Didymocarpoideae) with 160 species and 15 varieties (according to the GCR, Gesneriaceae Resource Centre, https://padme.rbge.org.uk/grc/, last accessed on 15, May 2025), which is primarily found in the mountainous regions of southwest and southern China (Möller et al., 2011; Kong et al., 2022; Xie et al., 2024a). Additionally, over a dozen species have been identified in Vietnam, Myanmar, Thailand, Japan, and India (Xie et al., 2024a). The morphological complexity and diversity of *Oreocharis* exhibit significant variations, leading to notable disparities in classification perspectives among scholars (Wang et al., 1990; 1998; Wang et al., 2010; Möller et al., 2011; 2016; 2025; Chen et al., 2014a, 2020). The taxonomic revision of *Oreocharis* involved the inclusion of species from several previously segregated genera, such as *Bournea* Oliver (Hooker, 1893), *Perantha* Craib (1918), *Tremacron* Craib (1918), *Ancylostemon* Craib (1920), *Isometrum* Craib (1920), *Dasydesmus* Craib (1920), *Opithandra* Burtt (1956), *Thamnocharis* Wang (1981), *Dayaoshania* Wang (1983a), *Schistolobos* Wang (1983b), *Deinocheilos* Wang (1986), and *Paraisometrum* W.T.Wang (Weitzman et al., 1998), and the acaulescent and rosette-forming species of *Briggsia* Craib (1920). Additionally, some species formerly described in the genus *Briggsia* or *Ancylostemon* were incorporated into the redefined *Oreocharis* (Chen et al., 2012; 2014b; Möller et al., 2011; 2014). Since Möller et al. (2011) newly delineated the genus *Oreocharis*, a total of 61 new taxa (including 60 species and one variety) have been published (last accessed on 15, May 2025, **Table 1**). However, currently, many descriptions of new species within the genus *Oreocharis* are primarily based on morphological characteristics (**Table 1**). Even when molecular data is used, it is often limited to a few DNA segments, such as ITS, *trnL-F*, and other regions (Chen et al., 2020b; Guo et al., 2018; Hu et al., 2023; Ling et al., 2020; 2022; Lv et al., 2021).

**Table 1:** Newly published taxa after Möller’s new delineation of the genus *Oreocharis*.

Scientific name	Chinese name	Type locality	Year	Reference
*Oreocharis dayaoshanioides* Yan Liu & W. B. Xu	齿叶瑶山苣苔	CHINA: Guangxi, Wuzhou	2012	Liu et al., 2012
*Oreocharis dimorphosepala* (W. H. Chen & Y. M. Shui) Mich. Möller	异萼直瓣苣苔	CHINA: Yunnan, Yuanyang	2012	Chen et al., 2012, 2014
*Oreocharis glandulosa* Y. H. Tan & J. W. Li	云南马铃苣苔	CHINA: Yunnan, Lancang	2013	Tan et al., 2013; 2014; Rossini and Freitas, 2014
*Oreocharis jinpingensis* W. H. Chen & Y. M. Shui	金平马铃苣苔	CHINA: Yunnan, Jinping	2013	Chen et al., 2013
*Oreocharis brachypoda* J. M. Li & Z. M. Li	短柄马铃苣苔	CHINA: Guizhou, Tongren	2015	Li and Li, 2015; Cai et al., 2020a
*Oreocharis hongheensis* (W. H. Chen & Y. M. Shui) Mich. Möller	红河短檐苣苔	CHINA: Yunnan, Honghe	2015	Cai et al., 2015; Möller, 2015.
*Oreocharis pilosopetiolata* L. H. Yang & M. Kang	毛梗马铃苣苔	CHINA: Guangdong, Huidong	2015	Yang et al., 2015a
*Oreocharis striata* F. Wen & C. Z. Yang	条纹马铃苣苔	CHINA: Fujian, Youxi	2015	Yang et al., 2015b
*Oreocharis synergia* W. H. Chen, Y. M. Shui & Mich. Moller	友谊马铃苣苔	CHINA: Yunnan, Yongsheng	2015	Chen et al., 2015
*Oreocharis tsaii* Y. H. Tan & J. W. Li	蔡氏马铃苣苔	CHINA: Yunnan, Menglian	2015	Tan et al., 2015
*Oreocharis baolianis* (Q. W. Lin) Li. H. Yang & M. Kang	保连马铃苣苔	CHINA: Fujian, Changting	2016	Lin, 2016; Yang et al., 2020
*Oreocharis curvituba* J. J. Wei & W. B. Xu	弯管马铃苣苔	CHINA: Guangxi, Guanyang	2016	Wei et al., 2016
*Oreocharis ninglangensis* W. H. Chen & Y. M. Shui	宁蒗马铃苣苔	CHINA: Yunnan, Ninglang	2016	Chen et al., 2016
*Oreocharis argyrophylla* W. H. Chen, H. Q. Nguyen & Y. M. Shui	银毛马铃苣苔	VIETNAM: Son La, Van Ho	2017	Chen et al., 2017a
*Oreocharis blepharophylla* W. H. Chen, H. Q. Nguyen & Y. M. Shui	毛缘马铃苣苔	VIETNAM: Son La, Van Ho	2017	Chen et al., 2017a
*Oreocharis caobangensis* T. V. Do, Y. G. Wei & F. Wen	高平马铃苣苔	VIETNAM: Cao Bang, Nguyen Binh	2017	Do et al., 2017
*Oreocharis crispata* W. H. Chen & Y. M. Shui	皱边马铃苣苔	CHINA: Guangxi, Quanzhou	2017	Chen et al., 2017b
*Oreocharis parviflora* Lei Cai & Z. K. Wu	小花马铃苣苔	CHINA: Yunnan, Lanping	2017	Cai et al., 2017
*Oreocharis purpurata* B. Pan, M. Q. Han & Yan Liu	紫纹马铃苣苔	CHINA: Hunan, Xinhua	2017	Han et al., 2017
*Oreocharis uniflora* L. H. Yang & M. Kang	单花马铃苣苔	CHINA: Guangdong, Huidong	2017	Yang et al., 2017
*Oreocharis zhenpingensis* J. M. Li, T. Wang & Y. G. Zhang	镇坪马铃苣苔	CHINA: Shaanxi, Zhenping	2017	Li et al., 2017
*Oreocharis duyunensis* Z. Y. Li, X. G. Xiang & Z. Y. Guo	都匀马铃苣苔	CHINA: Guizhou, Duyun	2018	Guo et al., 2018
*Oreocharis grandiflora* W. H. Chen, Q. H. Nguyen & Y. M. Shui	大花马铃苣苔	VIETNAM: Lao Cai, Sa Pa	2018	Chen et al., 2018
*Oreocharis longituba* W. H. Chen, Q. H. Nguyen & Y. M. Shui	长筒马铃苣苔	VIETNAM: Lao Cai, Sa Pa	2018	Chen et al., 2018
*Oreocharis ovata* L. H. Yang, L. X. Zhou & M. Kang	卵圆叶马铃苣苔	CHINA: Guangdong, Liannan	2018	Yang et al., 2018
*Oreocharis rufescens* D. J. Middleton	锈色马铃苣苔	VIETNAM: Lao Cai, Sa Pa	2018	Möller et al., 2018
*Oreocharis tribracteata* Bramley, H. J. Atkins & Mich. Möller	三苞马铃苣苔	VIETNAM: Ha Giang, Vi Xuyen	2018	Möller et al., 2018
*Oreocharis maximowiczii* var. *mollis* J. M. Li & R. Yi	密毛大花石上莲	CHINA: Fujian, Taining	2019	Yi et al., 2019
*Oreocharis odontopetala* Q. Fu & Y. Q. Wang	齿瓣粗筒苣苔	CHINA: Guizhou, Panzhou	2019	Fu et al., 2019a
*Oreocharis ovatilobata* Q. Fu & Y. Q. Wang	卵瓣马铃苣苔	CHINA: Guizhou, Panzhou	2019	Fu et al., 2019b
*Oreocharis panzhouensis* Lei Cai, Y. Guo & F. Wen	盘州马铃苣苔	CHINA: Guizhou, Panzhou	2019	Cai et al., 2019
*Oreocharis rubrostriata* F. Wen & L. E. Yang	红纹马铃苣苔	CHINA: Guangxi, Rongshui	2019	Yang et al., 2019
*Oreocharis tetraptera* F. Wen, B. Pan & T. V. Do	姑婆山马铃苣苔	CHINA: Guangxi, Hezhou	2019	Pan et al., 2019; Cai et al., 2020a
*Oreocharis aimodisca* Lei Cai, Z. L. Dao & F. Wen	滇东马铃苣苔	CHINA: Yunnan, Shizong	2020	Cai et al., 2020a
*Oreocharis argentifolia* Lei Cai & Z. L. Dao	银叶马铃苣苔	CHINA: Yunnan, Mengzi	2020	Cai and Dao, 2020b
*Oreocharis eriocarpa* W. H. Chen & Y. M. Shui	毛果马铃苣苔	CHINA: Yunnan, Wenshan	2020	Chen et al., 2020a
*Oreocharis flavovirens* Xin Hong	青翠马铃苣苔	CHINA: Gausu, Longnan	2020	Qin et al., 2020
*Oreocharis fulva* W. H. Chen & Y. M. Shui	褐毛马铃苣苔	CHINA: Yunnan, Yongde	2020	Chen et al., 2020a
*Oreocharis jasminina* S. J. Ling, F. Wen & M. X. Ren	迎春花马铃苣苔	CHINA: Hainan, Qiongzhong	2020	Ling et al., 2020
*Oreocharis lacerata* W. H. Chen & Y. M. Shui	羽裂马铃苣苔	CHINA: Yunnan, Yongde	2020	Chen et al., 2020a
*Oreocharis longipedicellata* Lei Cai & F. Wen	长梗马铃苣苔	CHINA: Yunnan, Malipo	2020	Cai et al., 2020a
*Oreocharis wenshanensis* W. H. Chen & Y. M. Shui	文山马铃苣苔	CHINA: Yunnan, Wenshan	2020	Chen et al., 2020a
*Oreocharis wumengensis* Lei Cai & Z. L. Dao	乌蒙马铃苣苔	CHINA: Yunnan, Yanjin	2020	Cai et al., 2020c
*Oreocharis reticuliflora* Li H. Yang & X. Z. Shi	网纹马铃苣苔	CHINA: Sichuan, Xuyong	2021	Yang and Shi, 2021
*Oreocharis wenxianensis* X. J. Liu & X. G. Sun	文县马铃苣苔	CHINA: Gausu, Wenxian	2021	Liu and Sun, 2021
*Oreocharis xieyongii* T. Deng, D. G. Zhang & H. Sun	解勇马铃苣苔	CHINA: Hunan, Huayuan	2021	Lv et al., 2021
*Oreocharis guangwushanensis* Z. L. Li & Xin Hong	光雾山马铃苣苔	CHINA: Sichuan, Nanjiang	2022	Li et al., 2022
*Oreocharis hainanensis* S. J. Ling & M. X. Ren	海南马铃苣苔	CHINA: Hainan, Dongfang	2022	Ling et al., 2022
*Oreocharis phuongii* T. V. Do	玄福马铃苣苔	VIETNAM: Thua Thien Hue, Nam Dong	2022	Le et al., 2022
*Oreocharis polyneura* Y. H. Tan, F. Wen & Y. X. Gong	多脉马铃苣苔	CHINA: Yunnan, Lancang	2022	Gong et al., 2022
*Oreocharis qianyuensis* Lei Cai, J. W. Yang & Q. Zhang	黔渝马铃苣苔	CHINA: Guizhou, Kaili	2022	Yang et al., 2022
*Oreocharis tianlinensis* R. C. Hu, W. B. Xu & Y. Feng Huang	田林马铃苣苔	CHINA: Guangxi, Tianlin	2022	Hu et al., 2022
*Oreocharis chenzhouensis* X. L. Yu, R. H. Tu & A. Liu	郴州马铃苣苔	CHINA: Hunan, Chenzhou	2023	Zhou et al., 2023
*Oreocharis oriolus* J. Hu & F. Wen	黄鹂马铃苣苔	CHINA: Yunnan, Ninglang	2023	Hu et al., 2023
*Oreocharis repenticaulis* X. K. Huang, P. Yang & Yan Liu	匍茎马铃苣苔	CHINA: Guangxi, Tianlin	2023	Huang et al., 2023
*Oreocharis thanhii* T. P. A. Tran, K. S. Nguyen & K. Tan	谭氏马铃苣苔	VIETNAM:Lai Chau, Sin Ho	2023	Tran et al., 2023
*Oreocharis wuxiensis* C. Xiong, F. Chen & F. Wen	巫溪马铃苣苔	CHINA: Chongqing, Wuxi	2023	Xiong et al., 2023
*Oreocharis yangjifengensis* F. Wen & B. Chen	阳际峰马铃苣苔	CHINA: Jiangxi, Guixi	2023	Li et al., 2023
*Oreocharis hapii* K. S. Nguyen, Aver. & C. W. Lin	黄峡马铃苣苔	VIETNAM: Quang Nam, Dai Loc	2024	Nguyen et al., 2024
*Oreocharis scutifolia* Z. Xie & H. H. Kong	盾叶马铃苣苔	CHINA: Yunnan, Dayao	2024	Xie et al., 2024a
*Oreocharis corallodiscoides* Huan C. Wang & Xi Li	珊瑚叶马铃苣苔	CHINA: Yunnan, Yimen	2025	Li et al., 2025

As the species of this genus are mainly distributed in China, their Chinese names are given here.

Advancements in sequencing technologies have revolutionized the field of taxonomy, facilitating access to genome-scale molecular data (Hong et al., 2023). In the case of *Oreocharis*, transcriptome sequencing has been performed for 110 species (Kong et al., 2022). However, despite the potential of this molecular data, it has not been fully utilized in the process of describing new species within the genus. Taxonomists face challenges in accessing and analyzing genomic-level information, especially for those with limited bioinformatics skills. Extracting orthologous single-copy genes from transcriptome assemblies typically requires command-line tools and familiarity with a Linux environment, which poses challenges for taxonomists lacking bioinformatics training. To address this, we provide a detailed and user-friendly guide (Obtaining_single-copy_data_guide.txt on Figshare, https://doi.org/10.6084/m9.figshare.29457638.v2) on obtaining single-copy data from transcriptomes. By making this procedure accessible, taxonomists will be better equipped to incorporate molecular information effectively into species descriptions, thereby enhancing our understanding of the diversity and evolution of *Oreocharis*.

In this study, we aim to demonstrate the importance of integrating both morphological and molecular data for accurately describing and classifying of new species within the genus *Oreocharis* through six newly collected samples (XieZ3609, XieZ3631, XieZ4055, XieZ4061, XieZ4065, XieZ4071) from Sichuan, Southwest China. By combining these two complementary sources of information, we aim to provide a more robust and comprehensive framework for future taxonomic studies in the Gesneriaceae family and beyond. Our findings will not only deepen our understanding of *Oreocharis* but also provide a model for incorporating molecular data into plant species descriptions, with broader implications for biodiversity and evolutionary studies. As technology continues to evolve, the synergy between morphological and molecular approaches will undoubtedly play a pivotal role in deepening our understanding of plant diversity and evolution.

## Materials and methods

### Morphological description and comparison

Morphological data were obtained from field collections of living intact individuals in their natural habitat. Detailed data on flowers, fruits, and vegetative structures, as well as information on habitat, GPS coordinates, and elevation, were recorded. Voucher specimens and living materials were then collected for subsequent study. Morphological measurements were conducted using a vernier caliper (Yantai Greenery Tools Co., Ltd., Yantai, China) to document diagnostic characters relevant to species delimitation. This was done to meticulously document specific characters deemed pertinent for the differentiation of species. Concurrently, digital photographs—especially close-up images of floral structures — were taken to capture the key morphological traits of the new species in detail (Xie et al., 2024a). Terminology used to describe morphological features followed Harris and Harris (1994) and the Flora of China (Wang et al., 1990; 1998).

After field investigations, specimen examinations at herbaria (e.g. IBSC, KUN, PE, etc.) were also essential for obtaining additional morphological and distribution data when available specimens existed. It should be noted that species formerly assigned to the genera *Bournea*, *Perantha*, *Tremacron*, *Ancylostemon*, *Isometrum*, *Dasydesmus*, *Opithandra*, *Thamnocharis*, *Dayaoshania*, *Schistolobos*, *Deinocheilos*, *Paraisometrum*, and the acaulescent, rosette-forming species of *Briggsia* are of special taxonomic relevance. Although these taxa were transferred to *Oreocharis* by Möller et al. (2011; 2014), divergent classification systems used by different herbaria (e.g., the Hutchinson system at IBSC, KUN, and IBK; the Engler system at PE and SZ) necessitate searching for specimens under their former generic names. Voucher specimens (including type materials) were deposited at herbarium of South China Botanical Garden (IBSC), Guangzhou, China. Additional examined specimens of *Oreocharis* were obtained from the herbaria CDBI, IBSC, KUN, PE and SM. As a supplement, digital specimens were examined through the JSTOR Global Plants web portal (https://plants.jstor.org/) and the National Plant Specimen Resource Center (www.cvh.ac.cn/index.php).

### Taxon sampling for molecular analyses

A total of 135 samples were used in this study, representing six newly collected samples, 106 previously described *Oreocharis* species (Supplementary Table 1 on Figshare, https://doi.org/10.6084/m9.figshare.28829327.v6), and six species from other genera of Gesneriaceae as outgroups: *Aeschynanthus buxifolius* Hemsl., *A. moningeriae* (Merr.) Chun, *Anna mollifolia* (W.T. Wang) W.T. Wang et K.Y. Pan, *Cyrtandra hawaiensis* C.B.Clarke, *Didymocarpus cortusifolius* (Hance) W.T. Wang and *Petrocodon dealbatus* Hance, selected based on previous phylogenetic analyses (Möller et al., 2011; Kong et al., 2022). During the field investigation, we meticulously collected one or more intact living plants. To protect the roots during transport, the lower portions of each plant were wrapped in moist mosses. If moss was unavailable, damp paper towels were used instead (Kong et al., 2022; Xie et al., 2024a). Prior to packing, photographs were taken and basic morphological observations and measurements were conducted. Subsequently, these living plants were sent to the greenhouse at the South China Botanical Garden, Chinese Academy of Sciences for cultivation and recovery. After recovery, fresh young leaves were harvested, cleaned of surface impurities, flash-frozen in liquid nitrogen with 50 mL EP tubes, and stored at -80°C (Kong et al., 2022; Xie et al., 2024a). All samples followed this procedure prior to transcriptome sequencing. Collection information is listed in Supplementary Table 1.

### RNA extractions and illumina sequencing

Frozen leaf tissues were sent to Novogene Corporation (Tianjin, China) for RNA extraction and transcriptome sequencing. Total RNA was extracted from each individual using the RNeasy Plant Mini Kit (Qiagen, Valencia, CA, USA), with on-column DNase I (RNase-free, TaKaRa, Dalian, China) treatment to eliminate genomic DNA contamination. RNA integrity and concentration were assessed using agarose gel electrophoresis and a 2100 Bioanalyzer (Agilent Technologies). Purified RNA was used for cDNA library construction, followed by 150 bp paired-end sequencing on an Illumina NovaSeq 6000 platform (Kong et al., 2022). All transcriptome data are available at NCBI (Kong et al., 2022; Xie et al., 2024a), and the newly generated raw reads are publicly available under NCBI BioProject accession number PRJNA1032259, with BioSample accession numbers SAMN47951770 to SAMN47951775 (https://www.ncbi.nlm.nih.gov/sra/PRJNA1032259).

### Reads filtering, 574 orthologs selection and 353 genes recognition

Quality control on the raw reads was performed to ensure the dependability of downstream analyses. In particular, fastp (Chen, 2023) was used to eliminate low-quality reads with parameter -g -q 5 -u 50 -n 15 -l 150 -overlap_diff_limit 1 -overlap_diff_percent_limit 10, and to obtain clean reads (Xie et al., 2024a). Orthologs identification was performed using GeneMiner (Xie et al., 2024b), based on a previously published dataset (https://doi.org/10.6084/m9.figshare.26927632.v1) containing 574 orthologous single-copy genes from 106 *Oreocharis* species, resulting in a new dataset (Dataset_1 on Figshare, https://doi.org/10.6084/m9.figshare.28829264.v2). A second dataset consisting 353 genes (Dataset_2 on Figshare, https://doi.org/10.6084/m9.figshare.28829282.v2) was generated using Easy353 with parameter -k1 31 -k2 41 -t1 5 -t2 5 -reference_number 100, based on the reference “353_ref_Gesneriaceae” downloaded using build_database.py (Zhang et al., 2022).

### Phylogenetic analysis

A total of 135 individuals (including outgroups) were included in the molecular phylogenetic analysis, representing 106 described *Oreocharis* species, six newly collected samples and six outgroups (Supplementary Table 1). Gene alignments were performed using MAFFT 7.525 (Nakamura et al., 2018) with default parameters, and ambiguous aligned regions were trimmed using Trimal 1.4.rev15 with the automated1 setting (Capella-Gutiérrez et al., 2009). Maximum likelihood (ML) trees were inferred for each gene separately using IQ-TREE 2.0.4 (Minh et al., 2020). The resulting gene trees were then used as input for species tree inference in ASTRAL-III 5.7.8 (Zhang et al., 2018), with node support values calculated using the local posterior probability (LPP) method (Sayyari and Mirarab, 2016), based on the 574 single-copy genes dataset and 353 genes dataset, respectively.

## Results

### Morphology

**Figure 1:**
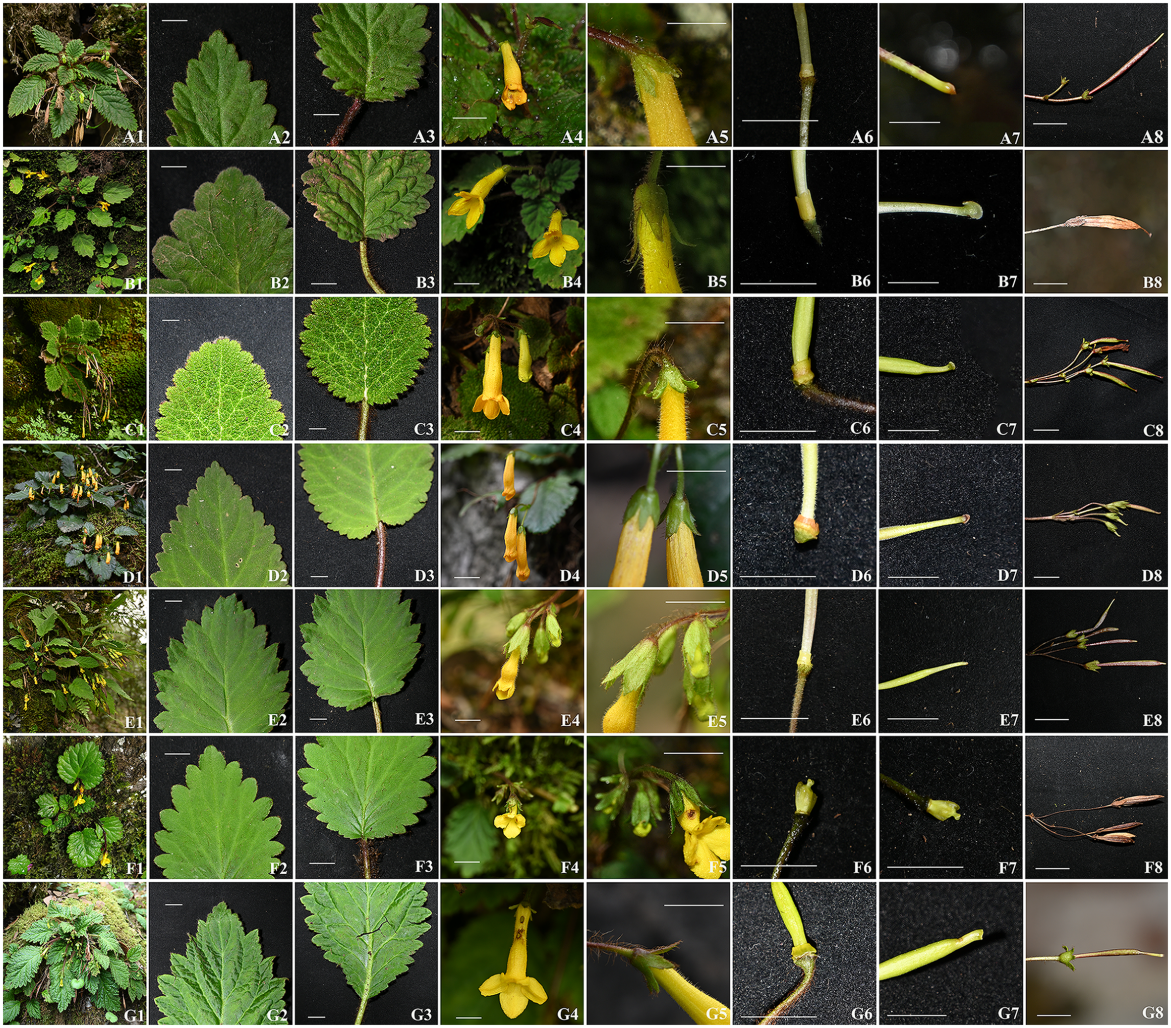
Morphological comparison among *Oreocharis convexa* **(A1–A8)**, *O. trichantha* **(B1–B8)**, *O. bullata* **(C1–C8)**, *O. saxatilis* **(D1–D8)**, *O. concava* **(E1–E8)**, *O. yanbianensis* **(F1–F8)**, and *O. gamosepala* **(G1–G8)**. **(A1, B1, C1, D1, E1, F1, G1)** Habit; **(A2, B2, C2, D2, E2, F2, G2)** Leaf blade apex; **(A3, B3, C3, D3, E3, F3, G3)** Leaf blade base; **(A4, B4, C4, D4, E4, F4, G4)** Flowers; **(A5, B5, C5, D5, E5, F5, G5)** Calyx; **(A6, B6, C6, D6, E6, F6, G6)** Disc; **(A7, B7, C7, D7, E7, F7, G7)** Stigma; **(A8, B8, C8, D8, E8, F8, G8)** Fruits. All scale bars represent 1 cm. Photographs: B4–B7 by Lihua Yang, others by Zhi Xie.

**Figure 2:**
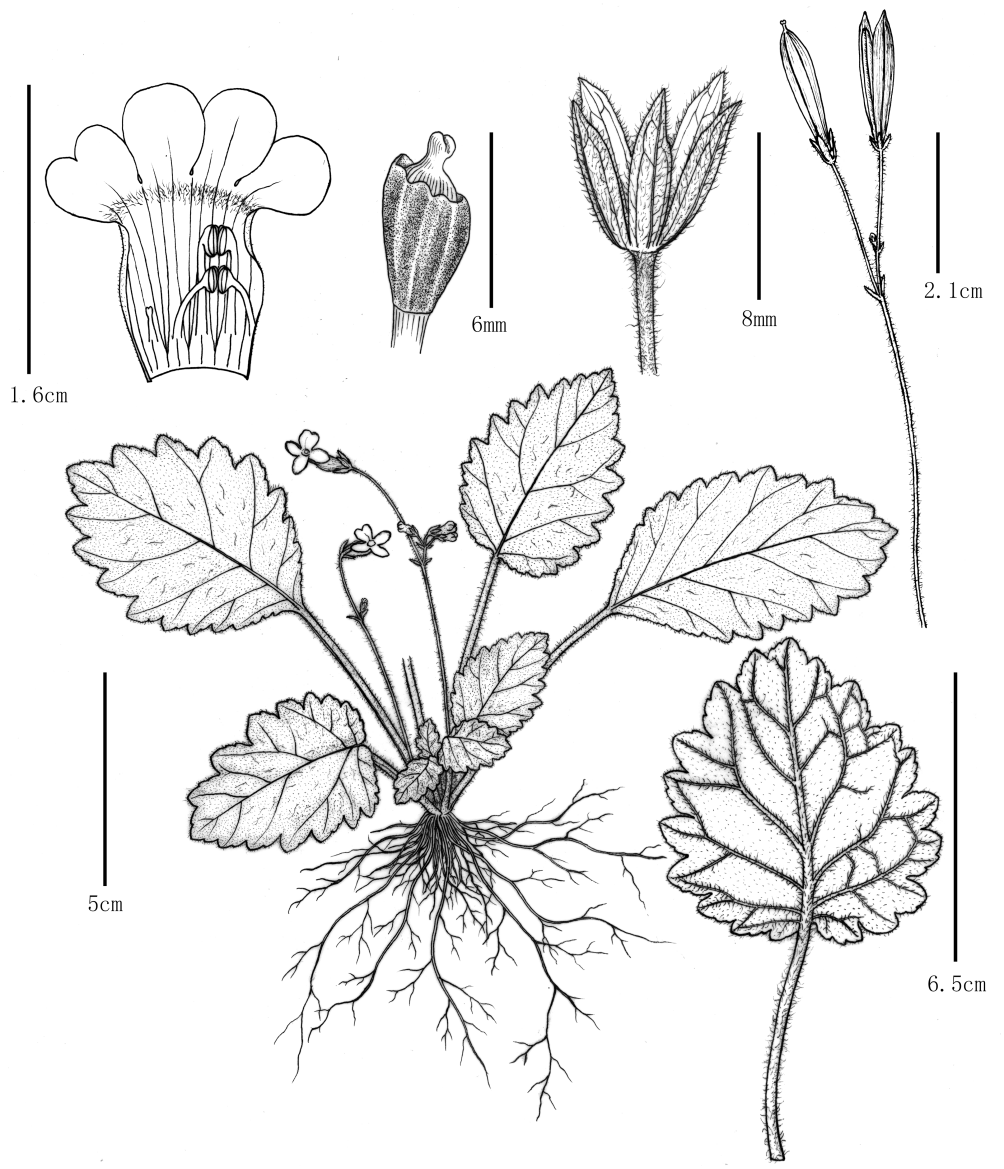
*Oreocharis yanbianesis* Z. Xie & H. H. Kong, sp. nov. Drawn by Yun-Xiao Liu.

**Figure 3:**
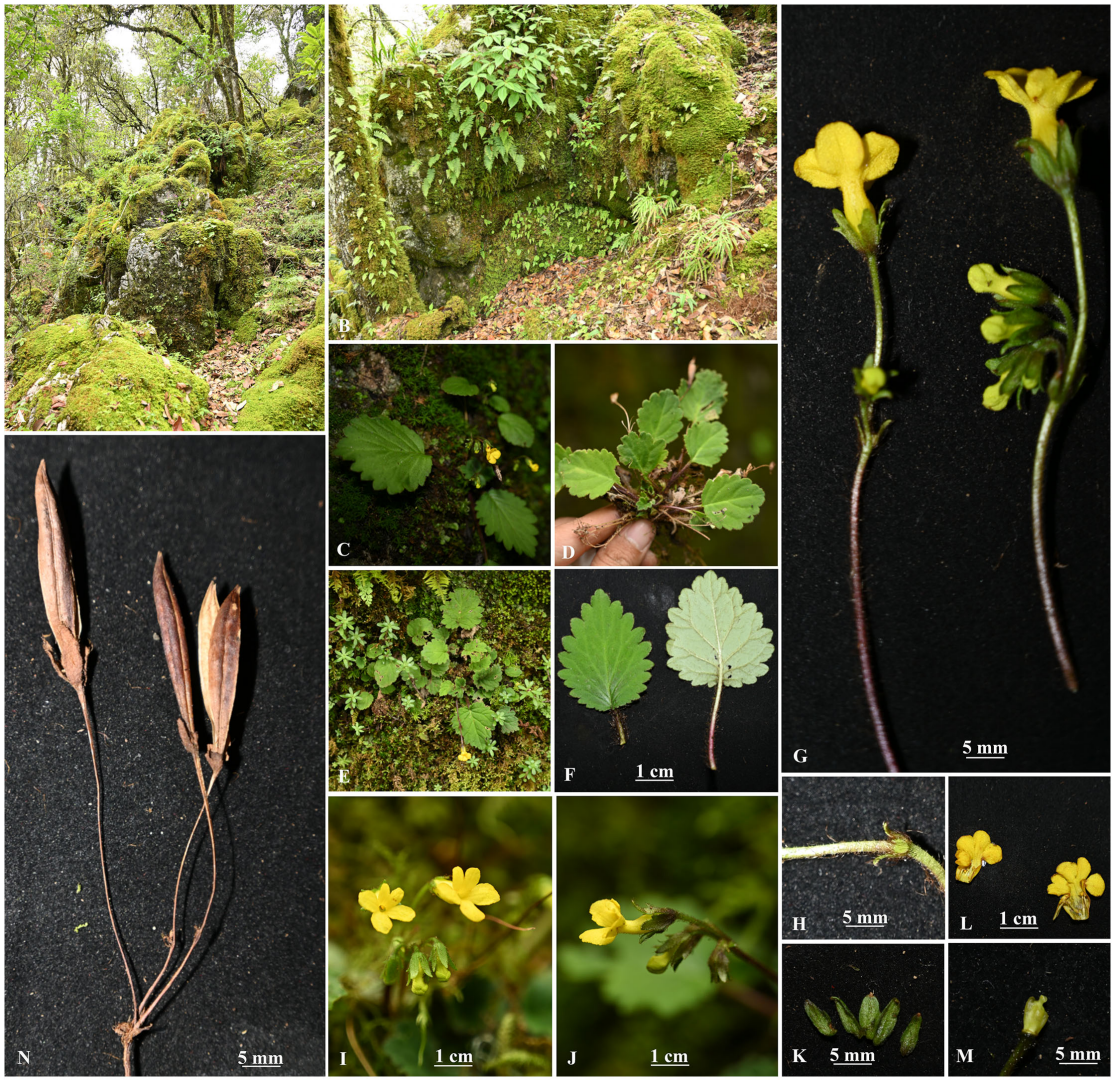
*Oreocharis yanbianesis* Z. Xie & H. H. Kong, sp. nov. **(A)** Habitat, the subtropical evergreen broad-leaved forests with *Schima argentea* E. Pritz. and *Quercus* spp.; **(B)** Population, rocks on limestone hillsides; **(C)** Habit; **(D)** individual without flower; **(E)** individual flowers; **(F)** Mature leaves: adaxially blade (left) and abaxially blade (right); **(G)** Cymes; **(H)** Bracts and pedicel; **(I)** Front view of flower; **(J)** Right side view of the flowers; **(K)** Calyx; **(L)** Opening corolla, showing stamens and staminode; **(M)** Disc, ovary and stigma; **(N)** Dehiscent capsule. Photos by Zhi Xie.

A detailed morphological comparison between the six newly collected samples and previously described *Oreocharis* species revealed several shared traits such as thickly chartaceous leaf blades and yellow flowers (**Figure 1**), and showed remarkable resemblance to several species, including *O. convexa* (Craib) Mich. Möller & A. Weber, *O. trichantha* (B. L. Burtt & R. A. Davidson) Mich. Möller & A. Weber, *O. bullata* (W. T. Wang & K. Y. Pan) Mich. Möller & A. Weber, *O. saxatilis* (Hemsl.) Mich. Möller & A. Weber and *O. concava* (Craib) Mich. Möller & A. Weber (**Figure 1**; **Table 2**). Morphologically, the species most similar to XieZ4071 is *O.trichantha* (**Figures 1**-**3**). However, it exhibits a throat-constricted, limp, distinctly two-lipped corolla with unequal lobes; the adaxial lip is obtuse, the abaxial lip rounded, and the pistil is shorter, 3.0 ± 0.4 mm. In contrast, *O. trichantha* possesses throat not constricted corolla, corolla slightly 2-lipped, all lobes subequal, obovate, and pistil longer, > 10 mm (**Figures 1**-**3** & **Table 2**). Further examination of herbarium specimens revealed that YLH710 was also collected from the same location, and its morphological traits were consistent with those of Xie4071.

**Table 2:** Morphological comparison among *Oreocharis convexa*, *O. trichantha*, *O. bullata*, *O. saxatilis*, *O. concava*, *O. yanbianensis* and *O. gamosepala*.

Characters	*O. convexa*	*O. trichantha*	*O. bullata*	*O. saxatilis*	*O. concava*	*O. yanbianensis*	*O. gamosepala*
Habitat	On rocks under the mountainous humid evergreen broad-leaved forests.	On rocks under the humid evergreen broad-leaved forests.	On rocks under forests or valleys.	On rocks under the humid evergreen broad-leaved forests.	Epiphytic to the tree trunks, or on rocks, under the humid evergreen broad-leaved forests.	On rocks under the mountainous broad-leaved forests.	On rocks under the mountainous broad-leaved forests.
Leaf blade							
Shape	Ovate.	Broadly ovate.	Ovate to ovate-rhombic.	Lanceolate to broadly ovate or oblong.	Broadly ovate to oblong ovate or elliptic.	Orbiculate rounded.	Ovate to oval.
Adaxial surface	White puberulent and sparsely rust-brown villous.	White puberulent and sparsely brown bristly-villous.	Densely puberulent.	Densely white puberulent and sparsely rust-brown villous.	Densely white puberulent and sparsely brown villous.	Rarely brown villous and sparsely white pubescent.	Sparsely brown villous and white pubescent.
Abaxial surface	White puberulent and sparsely rust-brown villous.	White puberulent.	Densely white puberulent.	White puberulent and pubescent, rust-brown villous.	Densely white pubescent, sparsely brown villous.	Rarely brown villous and sparsely white pubescent.	Sparsely brown villous.
Base	Oblique, rounded to cordate.	Rounded to cordate.	Broadly cuneate to rounded.	Oblique, cordate to cuneate.	Oblique, broadly cuneate to cordate.	Cordate to rounded.	Cuneate.
Margin	Double serrate to lobulate, lobes dentate to serrate.	Irregularly lobulate.	Double dentate, teeth obtuse.	Coarsely dentate-serrate or crenate to nearly lobulate.	Coarsely double dentate to double serrate or lobulate.	Serrate crenate.	Biserrate.
No. of Lateral veins	4 – 7 pairs.	4 or 5 pairs.	4 or 5 pairs.	4 – 7 pairs.	4 – 6 pairs.	4 – 6 pairs.	5 – 9 pairs.
Peduncle indumentum	Rust-brown villous and sparsely white puberulent.	Sparsely brown bristly-villous.	Spreading pubescent.	Brown villous and pale brown puberulent.	Pubescent and sparsely brown villous.	Densely brown villous.	Densely brown villous.
Bract	2, Linear to narrowly triangular.	2, lanceolate to ovate-lanceolate.	2, linear to narrowly triangular.	2, narrowly oblong to lanceolate or oblanceolate	2 – 4, narrowly oblong to obovate.	2, lanceolate.	2 – 4, linear.
Calyx							
Shape	5-lobed from below to above the middle.	5-lobed from middle.	5-lobed from middle.	5-lobed from below to near middle, rarely 5-sect from near base.	5-lobed from above middle.	5-sect nearly from base.	5-sect from below the middle.
Lobe shape	Lanceolate to ovate	Triangular.	Triangular.	Ovate to lanceolate.	Broadly triangular to ovate.	Lanceolate.	Ovate.
Margin	Dentate or entire	Sometimes denticulate.	Denticulate.	Dentate, rarely subentire.	Irregularly dentate.	Entire.	Regularly serrate.
Corolla limp							
Color and size	Orange to orange-yellow, 2.0 – 2.5 cm.	Yellow, 1.7 – 2 cm.	Yellow to orange, ca. 2.4 cm.	Yellow, 1.9 – 3.5 cm.	Orange to yellow, 2.2 – 2.8 cm.	Yellow, 1.2 – 2.0 cm.	Yellow, 1.8 – 3.2 cm.
Adaxial lip shape and size	Rounded, 2.0 – 4.5 × 2.0 – 3.5 mm.	Obovate, ca. 4 × 3 mm.	Indistinctly 2-lobed, ca. 4.5 × 5 mm.	Rounded, 1.0 – 3.0 × 1.0 – 3.0 mm.	Rounded, 1.5 – 3.0 × 4.0 – 4.5 mm.	Rounded, 1.8 – 2.1 × 2.0 – 2.4 mm.	Ovate, 3.2 – 3.6 × 0.9 – 1.6 mm.
Abaxial lip lobes shape and size	Equal, obovate, 4.5 – 6.0 × 3.0 – 5.0 mm.	Equal, obovate, ca. 4 × 3 mm.	Unequal, obovate-oblong to oblong, 5.0 – 6.5 × 2.5 mm.	Unequal, ovate-orbicular to broadly ovate, 4.0 – 10.0 × 3.0 – 6.5 mm.	Unequal, oblong to obovate-oblong, 4.0 – 7.0 × 4.0 – 5.5 mm.	Equal, rounded. 3.6 – 4.8 × 3.8 – 4.8 mm.	Equal, oval, 5.8 – 6.8 × 4.0 – 4.6 mm.
Stamens							
Filaments length	6 – 15 mm.	2 – 4 mm.	4.5 – 8.5 mm.	4.0 – 8.0 mm.	5.0 – 6.0 mm.	2.8 – 3.6 mm.	7.1 – 11.4 mm.
Staminode length	ca. 2.5 mm.	ca. 1 mm.	ca. 0.6 mm.	ca. 4 mm.	1.0 – 3.0 mm.	1.1 – 1.3 mm.	3.1 – 5.3 mm.
Ovary	Elliptic, glabrous.	Elliptic, glabrous.	Elliptic, glabrous.	Elliptic, densely white pubescent.	Elliptic, glabrous.	Globose, glabrous.	Elliptic, glabrous.
Disc	Yellow, ca. 1 mm, entire.	Yellow, ca. 2.2 mm, 5-denticulate, unequal.	Yellow, ca. 1.8 mm, entire, equal.	Orange, 1 – 1.5 mm, 5-lobed, equal.	Yellow, 2 – 5 mm, margin 5-lobed, equal.	Light yellow, 2.0 – 3.4 mm, margin 5-lobed, distinctly unequal.	Yellow, 1 – 2 mm, margin shallowly 5-lobed, equal.
stigma	Emarginate.	Disciform.	Disciform.	Disciform.	Emarginate.	Distinctly 2-lobed.	2-lobed.

**Figure 4:**
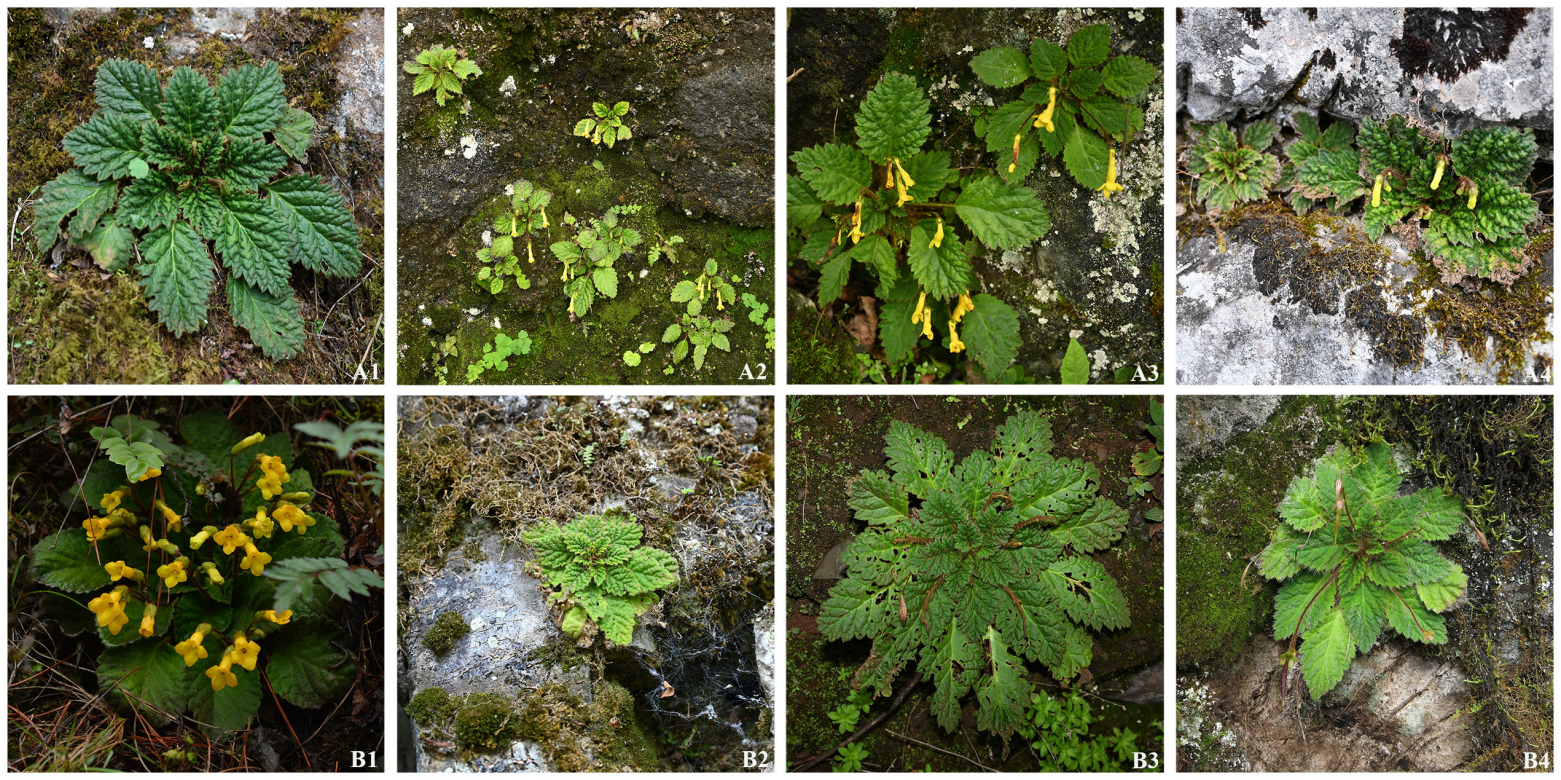
Morphological differences of *O. gamosepala* individuals between the same population **(A1–A4)** and different populations **(B1–B4)**. **(A1–A4)** were taken from the same locality of XieZ3631, Yanyuan, Sichuan, China by Zhi Xie; **(B1)** was taken from Zhaotong, Yunnan, China by Youpai Zeng; **(B2)**was taken from the same locality of XieZ3609, Yuexi, Sichuan, China by Zhi Xie; **(B3)** was taken from the same locality of YLH715, Yanyuan, Sichuan, China by Lihua Yang; **(B4)** was taken from the same locality of XieZ4061, Muli, Sichuan, China by Zhi Xie.

XieZ3631 resembles *O. gamosepala* (K. Y. Pan) Mich. Möller & A. Weber in floral morphology. Only minor differences in leaf size were observed between XieZ3631 and *O. gamosepala* (**Figure 1** & **Table 2**). Comprehensive observations of wild populations have demonstrated that leaf size varies significantly both among populations and between individuals within the same population (**Figure 4**). We also examined the holotype of *O. gamosepala* (Hanyuan expedition, 0427, 11 June 1978, SM 717900003)! and paratypes (Yanyuan expedition, 368, 20 June 1978, SM 717900003! Sichuan Economic Plant expedition of Liangshan, 3508, 28 June 1959, CDBI 0130063! CDBI 0130064! KUN 0206255! PE 00030696! PE 00030697! SM 717900020! SM 717900021)!. Further field investigations and specimen examinations confirmed that multiple collections — including Qinghai-Tibetan expedition 12613 (Qinghai-Tibetan expedition, Yanyuan, 30 July 1983, PE 01173170, PE 01173171, KUN 0206235), Qinghai-Tibetan expedition, 12116 (Qinghai-Tibetan expedition, Yanyuan, 20 July 1983, PE 01173173, PE 01173172, KUN 0206237), FCY2013016 (C. Y. Feng, Yanyuan, 13 October 2013, PE 02053062), YLH712 (H. H. Kong & L. H. Yang, Yanyuan, 18 August 2018, IBSC0882883, IBSC0882884), SCYY01 (H. H. Kong, L. H. Yang & B. F. Zhou, Yanyuan, 7 September 2017, IBSC), XieZ3609 (Z. Xie & M. Zhang, Yuexi, 27 June 2023, IBSC). XieZ3641 (Z. Xie & M. Zhang, Yanyuan, 1 July 2023, IBSC), XieZ4055 (Z. Xie, Yanyuan, 25 July 2024, IBSC), XieZ4061 (Z. Xie, Muli, 25 July 2024, IBSC) and XieZ4065 (Z. Xie, Yanyuan, 26 July 2024, IBSC) are all conspecific with *O. gamosepala*. These specimens exhibit consistent morphological features, with only slight variation across individuals (**Figure 4**).

YLH715 (H. H. Kong & L. H. Yang, Yanyuan, 18 August 2018, IBSC 0882896, IBSC 0882822), was previously identified as *O. concava* (Kong et al., 2022). However, it exhibits a cuneate leaf base, margin crenate to dentate or serrate; calyx 5-lobed, lobes equal, ovate, margin regularly dentate. These characteristics showed its similarity to *O. gamosepala*. In contrast, *O. concava* typically has oblique, broadly cuneate to cordate leaf base, margin coarsely double dentate to double serrate or lobulate; calyx 5-lobed, segments unequal, broadly triangular to ovate, margin irregularly dentate (**Figure 4B3**). These differences in leaf and calyx morphology cast doubt on the accuracy of the previous species identification. As flowering specimens and living plants were not observed, this identification remains provisional and should be treated with caution.

Moreover, in our prior study, YLH859 was misidentified and temporarily labeled as *O.* sp*5*. Upon revisiting the collection locality for further population observation, the sample was confirmed to match the diagnostic features *O. concava*. This species is characterized by broadly ovate to elliptic leaf blades, oblique to cordate leaf base; calyx 5-lobed from above middle; lobes unequal, broadly triangular to ovate; adaxial corolla lip rounded and abaxial corolla lip lobes oblong to obovate-oblong, and pistil glabrous (**Figure 1**; **Table 2**).

Furthermore, the specimens of lrh006 (R. H. Liang, Yuexi, 12 September 2006, PE 02053535, PE 02053533, PE 02053534, PE 02053521) were originally identified as *O. gamosepala*. However, upon examination, we found that they differ markedly from *O. gamosepala* in several morphological traits, including ovate leaf base, petiole sparsely white pubescent, corolla tubular, yellow to orange, adaxial lip extremely short, abaxial lip lobes ovate. These discrepancies indicate that the specimens were likely misidentified (**Table 2**).

Morphological comparisons led to the reidentification of XieZ3609, XieZ3631, XieZ4055, XieZ4061, and XieZ4065 as *O. gamosepala*, and YLH859 as *O. concava*. In addition, XieZ4071 and YLH710 are proposed to represent a new species, *Oreocharis yanbianensis* Z. Xie & H. H. Kong, sp. nov. (**Figures 2**, **3**). All taxonomic conclusions above are based solely on morphological evidence. However, due to the widespread hybridization, gene flow, and incomplete lineage sorting (ILS) in *Oreocharis*, relying solely on a limited set of morphological traits may compromise identification accuracy. Therefore, integrating molecular evidence is essential to clarify the systematic position and improve the robustness of taxonomic conclusions.

### Molecular analyses

A total of 114 taxa (including outgroups and two newly collected taxa) were included in the molecular phylogenetic analysis (Supplementary Table 1). Dataset_1 comprises 574 orthologous genes, which including 23.8% missing sites, and Dataset_2 consist of 353 low-copy nuclear genes, which including 0.62% missing sites.

**Figure 5:**
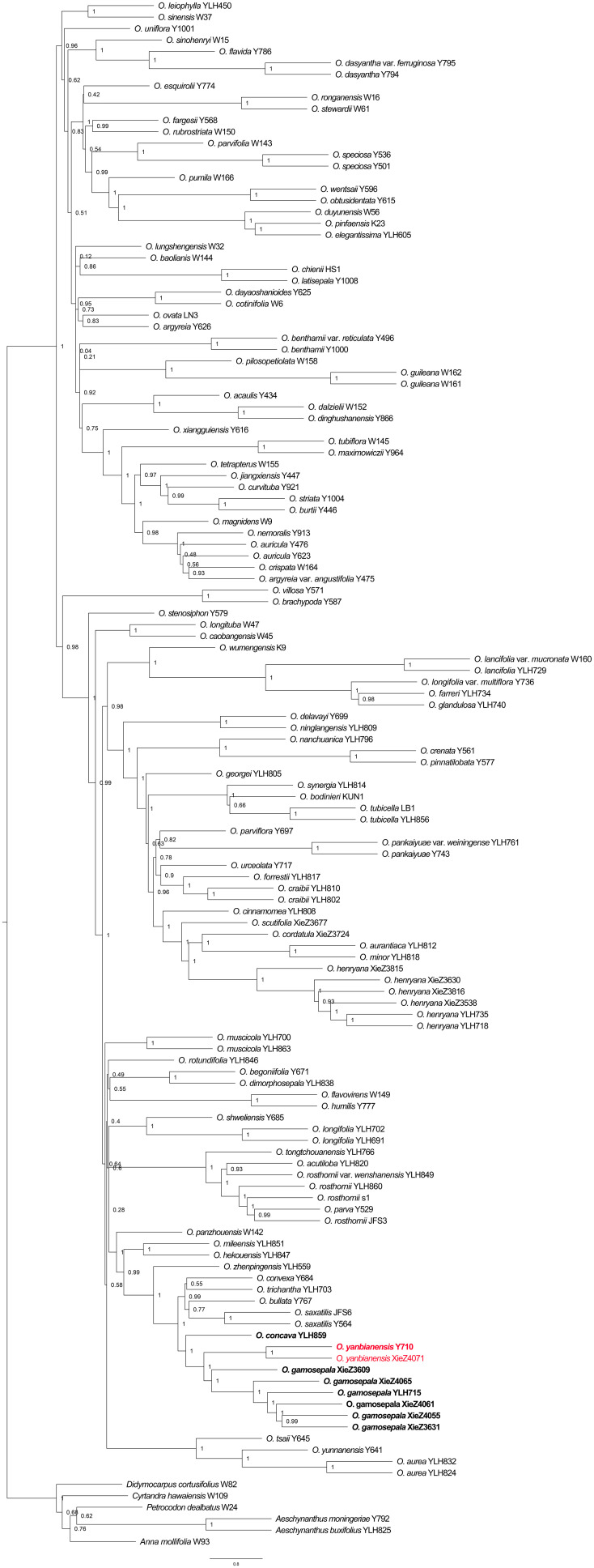
Phylogeny of *Oreocharis* based on the transcriptome dataset of 574 single-copy genes. The new species is shown in red, and the samples with species re-identification are marked in bold. The species tree was reconstructed by ASTRAL-III 5.7.8. Support for branches was evaluated with local posterior probability (LPP).

Phylogenetic analyses based on Dataset_1 strongly supported the clustering of *O. gamosepala*, *O. yanbianensis* sp. nov., *O. concava*, *O. saxatilis*, *O. bullata*, *O. trichantha* and *O. convexa* (LPP = 1; **Figure 5**), all of which share common morphological traits such as thickly chartaceous leaf blades and tubular yellow flowers (**Figure 1**). Within this clade, two samples — XieZ4071 and Y710 (YLH710 labeled on specimens) — were recovered as a maximum-supported monophyletic group (LPP = 1; **Figure 5**); both were collected from the same locality in Yanbian county and have been identified as *O. yanbianensis* sp. nov. based on morphological evidence. In addition, samples XieZ3609, XieZ4065, XieZ4061, XieZ4055, and XieZ3631, which were identified as *O. gamosepala*, clustered together with YLH715, also with strongest support (LPP = 1; **Figure 5**); support value for internal nodes within this subclade were also high (LPP ≥ 0.99; **Figure 5**). Furthermore, *O. saxatilis*, *O. bullata*, *O. trichantha*, and *O. convexa* formed another well-supported group (LPP = 0.99; **Figure 5**), within which *O. trichantha* and *O. convexa* appeared as sister species, albeit with relatively low support (LPP = 0.55; **Figure 5**).

**Figure 6:**
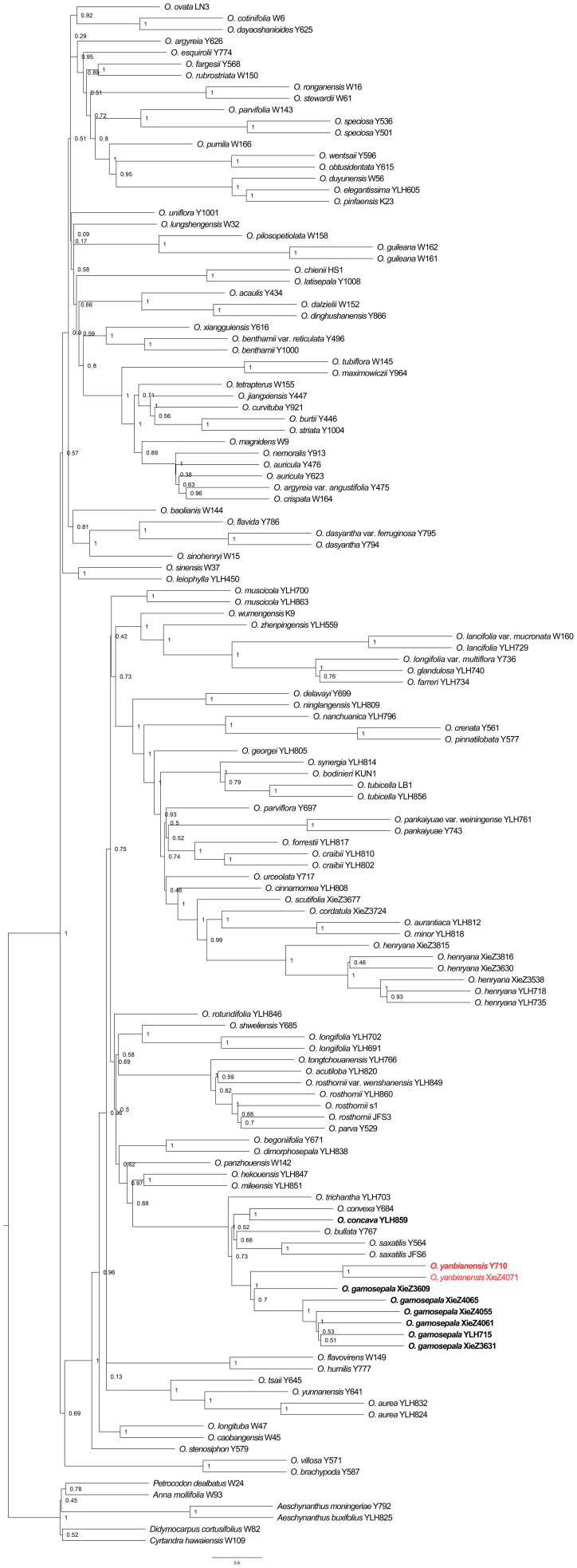
Phylogeny of *Oreocharis* based on the transcriptome dataset of 353 low-copy nuclear genes. The new species is shown in red, and the samples with species re-identification are marked in bold. The species tree was reconstructed by ASTRAL-III 5.7.8. Support for branches was evaluated with local posterior probability (LPP).

The phylogenetic analyses based on Dataset_2 also strongly supported the clustering of *O. gamosepala*, *O. yanbianensis* sp. nov., *O. concava*, *O. saxatilis*, *O. bullata*, *O. trichantha*, and *O. convexa* (LPP = 1; **Figure 6**). However, a notable difference is that several internal nodes within the *O. gamosepala* clade, which includes six samples exhibited lower support values (LPP ≤ 0.7; **Figure 6**). Additonally, *O. convexa* and *O. concava* clustered together with strong support (LPP = 1; **Figure 6**), with *O. trichantha* forming the basal lineage of this subclade. In previous studies, *O. zhenpingensis* J. M. Li, T. Wang & Y. G. Zhang was consistently resolved as the earliest-diverging lineage of a clade comprising several yellow-flowered species, e.g., *O. concava*, *O. saxatilis*, *O. bullata*, *O. trichantha* and *O. convexa* (Kong et al., 2022; Yang et al., 2022; Xie et al., 2024a). This placement was supported by both morphological traits and geographical distribution patterns (Liet al., 2017). However, in the species tree inferred from the 353 gene trees, *O. zhenpingensis* clustered with a strongly supported clade (LPP = 1, **Figure 6**) consisting of several purple-flowered species (*O. lancifolia* var. *mucronata* (K. Y. Pan) Mich. Möller & A. Weber, *O. lancifolia* (Franch.) Mich. Möller & A. Weber, *O. longifolia* var. *multiflora* (S. Y. Chen ex K. Y. Pan) Mich. Möller & A. Weber, *O. glandulosa* (Batalin) Mich. Möller & A. Weber, and *O. farreri* (Craib) Mich. Möller & A. Weber). This result is incongruent with previous morphological and biogeographic expectations and warrants further investigation.

By integrating morphological and molecular evidence, we identified XieZ3609, XieZ4065, XieZ4061, XieZ4055, XieZ3631 and YLH715 as *O. gamosepala* (**Figures 1**, **4**–**6**; **Table 2**), and recognized XieZ4071 and Y710 as representing a new species, *O. yanbianensis* Z. Xie & H. H. Kong sp. nov., which is illustrated and described below (**Figures 1**–**3**; **Table 2**). *O. yanbianensis* is morphologically most similar to *O. trichantha* (**Figure 1**; **Table 2**), but phylogenetically shows a closer relationship with *O. gamosepala* (**Figures 5**, **6**).

## Discussion

Frequent and substantial taxonomic revisions of *Oreocharis* have resulted in the shifting systematic positions of numerous species within this genus, accompanied by continuous changes in their scientific names (Möller et al., 2011; 2025; Fu et al., 2019c; Chen et al., 2020b). This adds a considerable layer of complexity to the studies involving this genus. Additionally, *Oreocharis* encompasses a wide range of plants exhibiting complex and variable morphological traits (Möller et al., 2011; Kong et al., 2022), which are often heavily influenced by environmental conditions and pollination pressures (Möller et al., 2011). Many species demonstrate significant variation in leaf shape, floral structure, and coloration. Notably, even within a single population, inter-individual trait variation can be substantial. Furthermore, striking morphological trait differences are often observed across developmental stages of the same individual (**Figure 4**). In contrast, convergent evolution driven by similar habitats and selective pressure can lead to analogous morphological traits in distantly related species. *O. yanbianensis* and *O. trichantha* exemplify this pattern in our present study (**Figures 1**, **4**, **6**).

For these reasons, misidentification of species is common in *Oreocharis*. For example, Qinghai-Tibetan expedition, 12116 (collected on 20 July 1983 from Dalin township — now Kalaba village, Baiwu township, Yanyuan county, Sichuan, China), comprises three duplicate specimens. One flowering specimen preserved in the Herbarium of Kunming Institute of Botany, Chinese Academy of Sciences (KUN) was identified as *Ancylostemon aureus* (Franch.) Burtt by Kaiyu Pan in 1986 (KUN0206237)!, while two fruiting specimens housed at the Institute of Botany, CAS (PE) and were labeled as *A. rhombifolius* K. Y. Pan (PE01173172! PE01173173)!, though without specified identifier and date. Interestingly, when *A. rhombifolius* K. Y. Pan sp. nov. was published in 1988, those specimens were not mentioned in the protologue (Pan, 1988). In 2017, we recollected this taxon (YLH715) from the same locality and initially misidentified it as *Oreocharis concava*, while YLH859 from Yongping, Yunnan was treated as potentially new species and labeled *O.* sp*5*. Meanwhile, specimens of YLH710 from Yanbian, Sichuan, was also misidentified as *O. concava* and included in previous phylogenetic analyses (Kong et al., 2022).

Since Wang et al. (2010) first constructed the phylogeny of *Oreocharis*, various molecular markers — such as *trnL-F* and ITS — have been employed in subsequent studies (Guo et al., 2018; Ling et al., 2020; 2022; Lv et al., 2021; Hu et al., 2023). In describing new species, some researchers incorporated newly sequenced samples into an existing molecular data matrix (e.g., Yang et al., 2020). However, many resulting phylogenies suffer from weak node support and potentially misidentified samples, failing to reflect the true phylogenetic relationship of this genus. This may be attributed to widespread hybridization, gene flow, and incomplete lineage sorting (ILS) in the genus (Kong et al., 2022). Furthermore, undersampling may have left many cryptic species unsampled and unsequenced, especially between poorly supported clades of *Oreocharis* (Kong et al., 2022).

In this study, we reconstructed the phylogeny of *Oreocharis* using both a 353 genes dataset and a 574 orthologous single-copy genes dataset based on transcriptome data. These analyses corrected several previous misidentifications of species. The inclusion or exclusion of a few species even altered tree topology, with some previously monophyletic groups becoming paraphyletic or polyphyletic. Y710 and YLH715 were previously classified as *O. concava*, demonstrating a strongly supported clade (ML = 100, Kong et al., 2022). However, in the present study, the inclusion of several *O. gamosepala* samples and XieZ4071 in the phylogenetic analysis led to their segregation into two distinct clades on the species tree. Y710 and XieZ4071 were clustered together with strong support (LPP = 1), indicating potential misidentification in these samples. This hypothesis was corroborated by evidence from morphological studies. We conducted multiple field expeditions over the past three years to verify these results through population-level observation and morphological comparison, combined with molecular evidence, which ultimately confirmed that YLH715 is *O. gamosepala*, YLH859 is *O. concava*, and Y710 represents an undescribed species.

Given these findings, describing new species solely based on limited morphological differences in *Oreocharis* is inadvisable. We advocate for comprehensive population-level studies and the integration of molecular evidence when describing new species. Such molecular evidence should not be limited to one or a few loci, but ideally include genome-scale datasets with a larger amount of information, such as the 353 genes dataset or the orthologous single-copy genes dataset. In our current phylogenetic reconstruction of *Oreocharis*, the 574 genes dataset appears robust, despite a few remaining topological conflicts and unresolved relationships.

Similar taxonomic issues caused by convergent evolution have been reported in other plant groups, emphasizing the broader relevance of our findings (Taha et al., 2023). Morphologically similar traits can evolve independently in unrelated taxa subjected to similar environmental pressures, such as drought, pollinator preference, or substrate type (Losos, 2011). In such cases, traditional morphology-based taxonomy alone can be misleading. A classic example lies in the striking resemblance between members of *Euphorbia* (Euphorbiaceae) and the cactus family Cactaceae. Although these groups are phylogenetically distant and geographically disjunct (*Euphorbia* in the Old World, Cactaceae in the New World), both have independently evolved succulent, spiny, columnar forms in response to arid conditions (Dorsey et al., 2013; Taha et al., 2023). These adaptations, although ecologically convergent, are not indicative of shared ancestry and have historically led to misclassification, especially in the absence of floral or molecular data (Guerrero et al., 2019).

Even within *Euphorbia*, recent phylogenomic work revealed at least 14 independent origins of drought-adaptive growth forms (Horn et al., 2012), underscoring the repeated emergence of misleading morphological similarity. Such findings illustrate the necessity of integrating genomic data—rather than relying on a limited number of morphological traits or DNA markers—especially in groups with high morphological plasticity or rapid ecological radiation. Consistent with this perspective, our study demonstrates how molecular evidence helped resolve misidentifications in *Oreocharis*. We advocate for a broader adoption of integrative taxonomy that combines morphology, molecular phylogenetics, ecology, and geography to achieve more robust and reproducible species delimitation.

### Taxonomy

*Oreocharis yanbianensis* Z. Xie & H. H. Kong, sp. Nov. (**Figures 2**, **3**).

### Type

CHINA, Sichuan: Panzhihua (攀枝花), Yanbian (盐边), Gesala (格萨拉), alt. 2,988 m, 27°08′20″ N, 101°19′45″ E, rocks on limestone hillsides in the subtropical evergreen broad-leaved forests with *Schima argentea* E. Pritz. and *Quercus* spp., flowering, 27 July 2024, Z. Xie, XieZ 4071 (holotype, IBSC! isotype, IBK)!.

### Diagnosis

This species resembles to *Oreocharis trichantha* in morphology, especially in leaf and flower (**Figures 1F1–F8**, **2**, **3**). However, it exhibits throat constricted corolla (vs. throat not constricted corolla), corolla limp 2-lipped, unequal, adaxial lip obtuse, abaxial lip rounded (vs. corolla slightly 2-lipped, all lobes subequal, obovate); pistil shorter, < 3 mm (vs. pistil longer, > 10 mm). On the basis of 574 single-copy genes analyses, *O. yanbianensis* showed closest relationships with *O. gamosepala*, and they formed a strongly supported sister group in phylogenetic tree.

### Description

Herb perennial and stemless, rhizomatous. Leaves basal, arrangement spiral; petiole sparsely brown villous, 0.8 – 6.5 cm long; leaf blade thickly chartaceous, orbiculate rounded, 3.4 – 7.0 × 2.2 – 4.5 cm; lateral veins 4–6 on each side of midrib; adaxially green, rarely brown villous and sparsely white pubescent, midrib veins slightly depressed, lateral veins distinct; abaxially light green, rarely brown villous and sparsely white pubescent, midrib and lateral veins distinct; base cordate to rounded, apex obtuse, margin serrate crenate. Inflorescence cymose, axillary, 2–4 per plant, 2–4 flowered per cyme. Peduncle 2 branched, 2–8 cm long, densely brown villous; bracts 2, lanceolate, 3.0 – 5.0 × 0.9 – 2.2 mm. Pedicel 1.5 – 6.5 cm long, densely brown villous. Calyx 3.8 – 5.0 mm long, 5-sect nearly from base; segments equal, lanceolate, 2.6 – 4.6 × 2.0 – 2.5 mm, margin entire, apex acuminate, adaxially sparsely brown villous, abaxially glabrous. Corolla tubular, yellow, 1.2 – 2.0 cm long, outside white pubescent and inside glabrous; tube cylindric, gradually narrowing towards mouth, 8–9 mm long, throat constricted, base of tube 3.6 – 4.8 mm in diam., middle of tube 2.2 – 2.9 mm in diam.; limb 2-lipped, 4.0 – 6.2 mm long, 9.5 – 13.6 mm wide; adaxial lip 2-lobed, 1.8 – 2.1 × 2.0 – 2.4 mm, apex obtuse; abaxial lip 3-lobed, segments equal, central lobe and lateral lobes rounded, apex rounded. 3.6 – 4.8 × 3.8 – 4.8 mm. Stamens 4, filaments glabrous; adaxial 2.8 – 3.4 mm long, adnate to corolla tube 1.5 – 1.8 mm from base; abaxial 3.0 – 3.6 mm long, adnate to corolla tube 1.0 – 1.4 mm from the base; anthers coherent in 2 pairs, included, 0.8 – 1.2 mm long, elliptic, 2-loculed, dehiscing longitudinally; staminode 1, usually not present, 1.1 – 1.3 mm long, adnate to corolla tube 1.1 – 1.2 mm from base. Disc ring-like, light yellow, 2.0 – 3.4 mm high, margin 5-lobed, unequal. Pistil glabrous, 0.25 – 0.36 cm long; ovary globose, 0.20 – 0.25 cm long, 1-loculed; style short, 0.05 – 0.09 cm long; stigma 1, 2-lobed. Capsule straight, widely elliptic, glabrous, 2–3 cm × 0.5 – 0.8 cm. Seeds tiny, elliptic, 0.50 – 0.56 × 0.10 – 0.12 mm.

### Distribution, habitat and phenology

This species is endemic to the Hengduan Mountains, southwest China: Sichuan (Panzhihua). On rocks of limestone hillsides in the subtropical evergreen broad-leaved forests with *Schima argentea* E. Pritz. and *Quercus* spp., alt. 2,900 – 3,000 m. Flowering is July to August, and fruiting is August to October.

### Conservation status

To date, only one population of the new species was discovered in the field, located at the type locality on the limestone hillside, comprising ca. 300 mature individuals and covering an area of approximately 10,000 m^2^ (100 × 100 m). This habitat is well-protected by the Lvshilin scenic spot, but is still influenced by various human activities through tourism. According to the IUCN Red List Categories and Criteria, the new species is hereby assessed as “Critically Endangered (CR)” (IUCN Standards and Petitions Subcommittee, 2024).

### Additional specimens examined (Paratypes)

CHINA, **Sichuan**: Panzhihua, Yanbian, Gesala, alt. 2,966 m, 27°8’22.39” N, 101°19’45.25” E, rocks on hillsides, flowering, 17 August 2018, H. H. Kong & L. H. Yang, YLH710 (IBSC0881928, IBSC0881929, IBSC0882485).

### Etymology

The species is named after its type locality.

### Vernacular name

In Chinese mandarin ‘Yán Biān Mǎ Líng Jù Tái’ (盐边马铃苣苔).

### Taxonomic key to selected species of *Oreocharis*

1. Ovary densely white pubescent. ……………………………….…*Oreocharis saxatilis*

1. Ovary glabrous or pubescent. …….….………………………2

2. Ovary globose. ……………………………………………….….…… *O. yanbianensis*

2. Ovary elliptic. ………………………………………….……3

3. Leaf base cuneate or broadly cuneate. ……………………….4

3. Leaf base rounded to cordate. … …………………………….5

4. Abaxial corolla lip lobes equal……………………………….…….… *O. gamosepala*

4. Abaxial corolla lip lobes unequal. ….………………….….…6

5. Corolla puberulent, disc ca. 1 mm, entire. …………………………….…. *O. convexa*

5. Corolla sparsely brown villous, disc ca. 2.2 mm, 5-denticulate. ………. *O. trichantha*

6. Leaf ovate to ovate-rhombic.………………………………………….…… *O. bullata*

6. Leaf broadly ovate to oblong ovate or elliptic. …………………………… *O. concava*

## Data Availability

The sequences used in this study have been deposited in the National Center for Biotechnology Information (NCBI) database. The transcriptome data of all samples used in this study are openly available from NCBI: https://www.ncbi.nlm.nih.gov/sra/PRJNA649046 & https://www.ncbi.nlm.nih.gov/sra/PRJNA1032259. The list with the collected locality and GenBank accession numbers of transcriptome data of all the samples (Supplementary Table 1, https://doi.org/10.6084/m9.figshare.28829327.v6), 574 orthologous single-copy genes matrix (Dataset_1, https://doi.org/10.6084/m9.figshare.28829264.v2), 353 low-copy nuclear genes matrix (Dataset_2 on Figshare, https://doi.org/10.6084/m9.figshare.28829282.v2) and the step by step guide on obtaining single-copy data from transcriptomes (Obtaining_single-copy_data_guide.txt on Figshare, https://doi.org/10.6084/m9.figshare.29457638.v2) can be found on Figshare (https://figshare.com/).
